# Kolmogorov–Smirnov and Cramér–von Mises tests for the *k*-sample problem with left-truncated and right-censored data

**DOI:** 10.1007/s10985-026-09713-1

**Published:** 2026-05-16

**Authors:** Adrián Lago, Juan Carlos Pardo-Fernández, Jacobo de Uña-Álvarez

**Affiliations:** 1 SiDOR Research Group, Department of Statistics and Operations Research, Universidade deVigo, Campus Universitario Lagoas-Marcosende, 36310 Vigo, Spain; 2 SiDOR Research Group, Department of Statistics and Operations Research, CITMAga, Universidade de Vigo, Campus Universitario Lagoas-Marcosende, 36310 Vigo, Spain

**Keywords:** Censoring, Cramér–von Mises, *K*-Sample problem, Kolmogorov–Smirnov, Truncation, 62G10, 62N03, 62G20

## Abstract

*k*-Sample versions of the Kolmogorov–Smirnov and Cramér–von Mises tests are proposed for data subject to left truncation and right censoring. Their asymptotic behaviour is studied and a bootstrap resampling plan is proposed to approximate the null distribution of the new tests. The performance of the tests with finite sample sizes is investigated in a simulation study, where the classical log-rank test is also considered. An illustration with a real dataset regarding unemployment times is discussed.

## Introduction

The main objective in many survival studies is to determine whether the target variable follows the same distribution across different populations. When dealing with more than two groups, it is also important to consider the problem of multiple comparisons and to use or design testing procedures that do not increase the probability of Type I error (similarly to the well-known ANOVA test for the comparison of means). The aim of this piece of research is to propose and study two tests with the aforementioned characteristic that allow for the comparison of distribution functions of $k \ge 2$ variables under truncation and censoring. For complete data, Kiefer (1959) proposed extensions of the classical two-sample Kolmogorov–Smirnov and Cramér–von Mises tests for the *k*-sample problem. Alternatives were studied in Birnbaum and Hall (1960) and Conover (1965). More recent papers are Zhang and Wu (2007) and Martínez-Camblor and de Uña-Álvarez (2009), where the practical performance of the tests is studied via simulations, in contrast to the older papers cited above.

Let us formulate our problem: consider *k* independent random variables, $X_1, \ldots , X_k$, and their respective cumulative distribution functions, $F_1, \dots , F_k$. We are interested in addressing the following hypothesis test

1 $$\begin{aligned} {\left\{ \begin{array}{ll} H_0: & F_1(t) = \ldots = F_k(t), \quad \text{ for } \text{ all } t \in \mathbb {R} \\ H_1: & F_j(t) \not = F_l(t), \; \text{ for } \text{ some } j,l \in \{1, \dots , k\} \text{ and } \text{ some } t \in \mathbb {R}. \end{array}\right. } \end{aligned}$$

That is, the null hypothesis states that the *k* distribution functions are equal and we will dismiss such a statement if we find significant differences between, at least, two of the distribution functions.

The object of study in this paper is left-truncated and right-censored data, which frequently appear in follow-up studies (Hyde 1977), clinical trials (Lagakos et al. 1988; Tassie et al. 2002; Asgharian et al. 2002), econometric studies (de Uña-Álvarez and Iglesias-Pérez 2010), and many other applied fields. Broadly speaking, data are censored when it is not possible to record the exact time to event of every observed individual. It is a common issue in Survival Analysis, where the main goal is to study the time until a specific event, usually called failure time. In particular, those that are only known to fail after the recorded time are said to be right-censored. Furthermore, it may also happen that data are left-truncated as a result, for example, of a cross-sectional sampling scheme or delayed entry times; that is, some individuals will be excluded of the study (thus not observed), when the event date precedes the entry-into-study date. In other words, individuals are not observed until a certain sampling condition is satisfied. Truncated data come from a biased sampling method and induces, among other issues, biased estimators if not treated properly, so that techniques for adequate inferences with left-truncated and right-censored data need to be developed in order to avoid misleading results.

There exists a broad literature addressing the comparison of distributions of random variables subject to right censoring. For instance, the Kolmogorov–Smirnov and Cramér–von Mises-type tests for goodness-of-fit with right-censored data proposed in Koziol and Green (1976) were extended to the two-sample problem in Schumacher (1984). Well-known methods in Survival Analysis are the rank-based tests, such as the log-rank test (see Mantel 1966 and Peto and Peto 1972) for two or more samples, and the conditional rank test in Neuhaus (1993), proposed for the two-sample problem. The literature for left-truncated data is much scarcer, since the main approach to address the comparison of distributions are the aforementioned rank-based tests properly adapted to left truncation. However, these type of tests are known for not being omnibus, thus they might be unable to detect the alternative hypothesis (see Section 7.3 in Klein and Moeschberger 2003). In contrast, omnibus tests for left-truncated data were studied in Lago et al. (2025), where a Kolmogorov–Smirnov-type test is proposed for the two-sample problem, and Lago et al. (n.d.), where a density-based test is studied to address the *k*-sample problem. The *k*-sample problem with left-truncated and right-censored data has not been studied deeply, being the rank-based tests, like the classical log-rank or the log-rank-type alternative in Ning et al. (2010), the main technique to tackle it. The goal of this article is to propose two tests that take left truncation and right censoring into account and are omnibus alternatives to the classical rank-based tests.

The rest of the paper is organised as follows. Section 2 contains the notation used throughout the manuscript and the estimator of the distribution function. New Kolmogorov–Smirnov and Cramér–von Mises-type tests for the *k*-sample problem with left-truncated and right-censored data are proposed in Sect. 3. Their asymptotic behaviour is studied as well and, due to the difficulty of application in practice of the null asymptotic distribution, a bootstrap resampling plan is proposed to approximate the null distribution of the tests in Sect. 3.2. The practical performance of the proposed methods is studied in Sect. 4 in a simulation study. Calibration results are shown first, and then the new proposals are compared to weighted rank-based tests in different simulation scenarios. Section 5 contains an illustration of the performance of the proposed tests with a real dataset regarding unemployment times. The main conclusions obtained in this paper are collected in Sect. 6 and the proofs of the theoretical results are displayed in Appendix A.

## Estimation with left-truncated and right-censored data

Let $j \in \{1, \ldots , k\}$. Let $U_j$ and $C_j$ denote the truncation and censoring random variables, respectively. Let us assume that the pair $(U_j, C_j)$ is independent of $X_j$, but we will allow for $U_j$ and $C_j$ to be dependent. Moreover, following the steps in Wang (1991), in order to avoid identifiability problems, we will assume that $U_j \le C_j$ with probability one. Under right censoring, $X_j$ is not fully observable; instead, values from $Y_j = \min \{ X_j, C_j \}$ are recorded, along with the indicator of the censoring status $\Delta _j = \mathbb {I} \left[ X_j \le C_j \right]$. The random variable $\Delta _j$ takes the value 1 if the event of interest is completely observed and 0 otherwise. Moreover, under left truncation, some of the individuals that experience the event of interest are not observed at all; only those such that $U_j \le X_j$ can be sampled. As a consequence, since data are subject to left truncation and right censoring, we will observe values from the random triplet $(U_j,Y_j, \Delta _j) \mid U_j \le X_j$. The sampling condition is sometimes given by $U_j \le Y_j$ in the left truncation and right censoring framework (for instance, in Gijbels and Wang 1993). However, the assumption $\mathbb {P}(U_j \le C_j)=1$ allows to rewrite it only in terms of the truncation and target variables. Let us denote by $G_j$ and $W_j$ the cumulative distribution functions of $U_j$ and $Y_j$, respectively and let $S_j = 1 - F_j$ be the survival function of $X_j$. In the sequel, the function $S_j$ will be assumed to be continuous.

Let $\left( U_{j1}, Y_{j1}, \Delta _{j1} \right) , \ldots , \left( U_{jN_j}, Y_{jN_j}, \Delta _{jN_j} \right)$ be an iid sample of size $N_j$ from $(U_j,Y_j, \Delta _j)$. Due to left truncation, the sample to work with is of the form $\left( U_{j1}, Y_{j1}, \Delta _{j1} \right) , \ldots , \left( U_{jn_j}, Y_{jn_j}, \Delta _{jn_j} \right)$. Under left truncation (with or without right censoring), the sample size is a random variable and, conditioned to it, the observed individuals are still independent. By the law of large numbers,

$$\begin{aligned} \frac{n_j}{N_j} \longrightarrow \gamma _j = \mathbb {P} \left( U_j \le X_j \right) , \end{aligned}$$

as $N_j \longrightarrow \infty$, where $\gamma _j$ is the so-called nontruncation probability. Of course, the lower $\gamma _j$ is, the more individuals are not observed because of truncation, for a fixed $N_j$.

Ignoring the left-truncated and/or right-censored nature of a dataset yields inconsistent estimators of the cumulative distribution function. The empirical distribution function evaluated over the observed values underestimates the target distribution at the left tail due to the left-truncation effect. On the other hand, ignoring right censoring leads to an overestimation of the target distribution at the right tail. It is then important to employ an estimator that overcomes the problems caused by both left truncation and right censoring simultaneously.

Consider the sample $\left( U_{j1}, Y_{j1}, \Delta _{j1} \right) , \ldots , \left( U_{jn_j}, Y_{jn_j}, \Delta _{jn_j} \right)$. Let $0< x_{j1}< \ldots < x_{jm_j}$ be the observed times to event, $d_{ji} = \# \{ Y_{jl} \mid Y_{jl} = x_{ji}, \; \Delta _{jl} = 1, l= 1,\ldots , n_j \}$ the number of events that take place at time $x_{ji}$ and $r_{ji} = \# \{ (U_{jl},Y_{jl}) \mid U_{jl} \le x_{ji} \le Y_{jl}, l= 1,\ldots , n_j \}$ the count of individuals at risk at time $x_{ji}$. Then, the cumulative distribution function estimator of $F_j$ is given by (Tsai et al. 1987)

2 $$\begin{aligned} F_{jn_j}(t) = 1 - \prod _{x_{ji} \le t} \left( 1 - \frac{d_{ji}}{r_{ji}} \right) . \end{aligned}$$

The previous estimator has been deeply studied in the literature (Wang 1991; Lai and Ying 1991; Gijbels and Wang 1993; Gross and Lai 1996b, and Zhou and Yip 1999). Without left truncation, (2) reduces to the Kaplan–Meier estimator (Kaplan and Meier 1958) for purely right-censored data. Analogously, without right censoring, (2) reduces to the Lynden-Bell estimator (Lynden-Bell 1971) for left-truncated data. Note that (2) only assigns positive mass to uncensored times, just like the Kaplan–Meier estimator does. We can think of (2) as the distribution function of a discrete random variable that takes the uncensored values of the sample with the probability given by the jumps of the estimator. This implies, in particular, that (2) is a stepwise, non-decreasing, right-continuous function. In particular, the stepwise property of the estimator will be exploited for the computation of the test statistics proposed in Sect. 3.

The estimation of the distribution function of the truncation random variable, $G_j$, is formally equal to that with only left-truncated data. The estimator is given by (Wang 1991)

3 $$\begin{aligned} G_{jn_j}(t) = \left( \sum _{l=1}^{n_j} S_{jn_j}(U_{jl})^{-1} \right) ^{-1} \sum _{i=1}^{n_j} S_{jn_j}(U_{ji})^{-1} \mathbb {I}\left[ U_{ji} \le t \right] , \end{aligned}$$

where $S_{jn_j}(t) = 1 - F_{jn_j}(t)$. The asymptotic properties of (3), such as the strong point consistency of $G_{jn_j}$ as an estimator of $G_j$, were studied in Wang (1991) and Shen (2003), where the problem of estimating $\gamma _j$ is also addressed (see also Shen 2005). Finally, the distribution function of the censoring random variable is difficult to estimate due to the possible dependence between the truncation and censoring random variables. To circumvent this issue, one could use the residual censoring time with a few more assumptions (see Sect. 3.2 for more details). Alternatively, the direct estimation of the censoring distribution function is not possible without assuming restrictive smoothing conditions (see Gross and Lai 1996a).

For a general random variable with distribution function *H*, let $a_{H} = \inf \{ t \mid H(t) > 0\}$ and $b_{H} = \sup \{ t \mid H(t) < 1\}$ be the lower and upper bounds of such a variable. Then, $a_{G_j}$ and $a_{F_j}$ denote the lower bounds of the supports of $U_j$ and $X_j$, respectively, and $b_{G_j}$ and $b_{W_j}$ are the upper endpoints of the supports of $U_j$ and $Y_j$, respectively. In order to obtain consistent estimators of $G_j$ and $F_j$, we need to assume that $a_{G_j} \le a_{W_j}$ and $b_{G_j} \le b_{W_j}$ (Gijbels and Wang 1993). Since $U_j \le C_j$ with probability one, one has $a_{G_j} \le a_{R_j}$ and $b_{G_j} \le b_{R_j}$, where $R_j$ stands for the distribution function of $C_j$; thus, since $Y_j = \min \{X_j,C_j\}$, the previous conditions reduce to

4 $$\begin{aligned} a_{G_j} \le a_{F_j} \quad \text{ and } \quad b_{G_j} \le b_{F_j}. \end{aligned}$$

The violation of the first condition above would imply that there is a part of the support of the target variable that is unobservable due to the left truncation, so that the distribution function cannot be fully estimated. That is a typical assumption made in the left truncation framework (see, for example, Woodroofe 1985, and Chao and Lo 1988). The second condition in (4) is needed to ensure that the left truncation distribution $G_j$ can be identified. The main results will be established on an interval with upper limit $b_j$, with $b_j < b_{W_j}$, thus taking right censoring effects into account.

In order to obtain the asymptotic results, Corollary 1 in Gijbels and Wang (1993) will be needed. For the sake of clarity, we reproduce it here. Let us assume that $X_j$ is continuous, (4) holds, and let $b_j < b_{W_j}$. Then, the process $\sqrt{n_j} \left( F_{jn_j}(t) - F_j(t) \right)$ converges weakly to a centred Gaussian process in $\mathbb {D}[a_{F_j},b_j]$ with covariance structure given by

5 $$\begin{aligned} \Gamma _j(t,s) = S_j(t) S_j(s) \int _{a_{F_j}}^{t \wedge s} \frac{dH^1_j(z)}{C_j(z)^2}, \end{aligned}$$

for $$t,s \in [a_{F_j},b_j]$$, where $$t \wedge s = \min \{t,s\}$$,

6 $$\begin{aligned} H^1_j(t) = \mathbb {P} \left( Y_j \le t, \Delta _j=1 \mid U_j \le X_j \right) = \gamma _j^{-1} \int _{a_{F_j}}^t \mathbb {P}\left( U_j \le z \le C_j \right) dF_j(z), \end{aligned}$$

which is the subdistribution function of $Y_j$ when $\Delta _j=1$, and

$$\begin{aligned} C_j(t) = \mathbb {P} \left( U_j \le t \le Y_j \mid U_j \le X_j \right) = \gamma _j^{-1} \mathbb {P} \left( U_j \le t \le C_j \right) S_j(t). \end{aligned}$$

Finally, for a better understanding of the asymptotic results in the next section, it is important to note that the process $\sqrt{n_j} \left( F_{jn_j}(t) - F_j(t) \right)$ is identically equal to zero for $t \notin [a_{F_j}, b_{F_j}]$.

## Kolmogorov–Smirnov and Cramér–von Mises-type tests

### The proposed test statistics and asymptotic results

The original Kolmogorov–Smirnov test (Smirnov 1939) was developed exclusively for the two-sample problem. There exist many different ways to extend the test statistic to the *k*-sample problem, like the ones described in Kiefer (1959) and Birnbaum and Hall (1960). In this paper, we will employ the statistic studied in detail for complete data in Kiefer (1959), given by

7 $$\begin{aligned} D_{KS} = \sup _{t \in \mathbb {R}} \sum _{j=1}^k n_j \left( F_{jn_j}(t) - F_n(t) \right) ^2, \end{aligned}$$

where $F_n$ is an estimator of the common distribution function under the null hypothesis, say *F*. There are different possibilities for the choice of $F_n$, but the one employed in this piece of research will be

8 $$\begin{aligned} F_n(t) = \sum _{j=1}^k p_j F_{jn_j}(t), \quad \text{ where } \; \sum _{j=1}^k p_j= 1 \; \text{ and } \; p_j >0, \; \text{ for } \text{ all } \; j \in \{1, \ldots , k\}, \end{aligned}$$

where $n = \sum _{j=1}^k n_j$. That is, $F_n$ is a weighted average of the estimators in each sample. In the simulation section and in the real data illustration, we will use the natural choice $p_j = n_j/n$ for $j \in \{1, \ldots , k\}$, which assigns more weight to the largest samples, namely, the ones containing more information. Note that, without censoring and truncation, this choice of $p_j$ yields the empirical cumulative distribution function of the pooled sample. Moreover, with this choice of $F_n$ as defined in (8), we are, indeed, dealing with an extension to the *k*-sample problem of the well-known two-sample Kolmogorov–Smirnov statistic proposed in Smirnov (1939). Indeed, if $k=2$, it is immediate to see that

$$\begin{aligned} D_{KS} = \left( n_1 p_2^2 + n_2 p_1^2 \right) \left( \sup _{t \in \mathbb {R}} \vert F_{1n_1}(t) - F_{2n_2}(t) \vert \right) ^2. \end{aligned}$$

One may wonder why not to merge the *k* samples and then employ the distribution function estimator defined in (2). Although that might seem a more natural proposal, it might not be a good estimator of the distribution in the presence of left truncation and right censoring. The problem lies in the nature of the estimator of the distribution function. Let us consider two time-to-event variables $X_1$ and $X_2$ left truncated by $U_1$ and $U_2$, respectively. Suppose, without loss of generality, that both $U_2$ and $X_2$ take larger values than $X_1$ and the supports of $X_1$ and $X_2$ are far apart from each other. The distribution function estimator of the merged samples would assign almost all of the weight to the times to event of $X_1$, which would yield an inconsistent estimation of the mixture distribution. The same inconsistency is expected when $X_1$ and $X_2$ are right censored by $C_1$ and $C_2$, respectively, if the distributions of $X_1$ and $X_2$ are far apart and $C_2$ takes values substantially larger than those of $X_1$. In this case, the risk sets of the event times from $X_1$ are incorrectly increased, which yields an underestimation of the underlying mixture distribution function. The comments above extend to data subject to left truncation and right censoring, so an estimator of the *k* merged samples is ruled out. Note that this does not occur with complete data, since the empirical distribution function of the merged samples can be obtained from a weighted average of the estimators in each sample with appropriate weights depending only on the sample sizes.

Due to the stepwise shape of the estimators $F_{jn_j}$, the Kolmogorov–Smirnov test is a maximum rather than a supremum, since it is attained in one of the uncensored event times of the samples. Note that the supremum in (7) coincides with the maximum computed on the interval $[\widehat{a},\widehat{b}]$, where

9 $$\begin{aligned} \widehat{a} = \min _{i,j} \{Y_{ji}, \Delta _{ji}=1 \} \qquad \text{ and } \qquad \widehat{b} = \max _{i,j} \{ Y_{ji} , \Delta _{ji}=1\}, \end{aligned}$$

namely $\widehat{a}$ and $\widehat{b}$ are the minimum and maximum uncensored observed event times, respectively. Note that the statistic in (7) is nonnegative and large values of $D_{KS}$ give evidence that, at least, two distribution functions are different from each other. In other words, the null hypothesis is rejected when $D_{KS}$ takes large values. The main goal of this piece of research is to determine when one can state that the test statistic takes a large value. In order to do that, the asymptotic distribution (see Theorem 1 and Corollary 1) is studied and a bootstrap resampling plan (see Sect. 3.2) is proposed to approximate the null distribution of the test statistic.

Moreover, we propose an adaptation of the *k*-sample Cramér–von Mises test, also defined in Kiefer (1959), to data subject to left truncation and right censoring. The test statistic is given by

10 $$\begin{aligned} D_{CvM} = \sum _{j=1}^k n_j \int \left( F_{jn_j}(t) - F_n(t) \right) ^2 dF_n(t), \end{aligned}$$

where the integral is defined over the whole real line and $F_n$ was defined in (8). Similarly to the case of the Kolmogorov–Smirnov-type test defined above, since $F_n$ is a discrete distribution with mass on the observed uncensored event times, the statistic (10) is actually a sum over a finite set of points contained in the interval $[\hat{a}, \hat{b}]$. As for the Kolmogorov–Smirnov test, large values of the Cramér–von Mises test suggest that the null hypothesis should be rejected. The proposed Cramér–von Mises-type test reduces to the classical two-sample Cramér–von Mises test when two distributions are compared. Indeed, setting $k=2$ yields

$$\begin{aligned} D_{CvM} = (n_1p_2^2 + n_2p_1^2) \int \left( F_{1n_1}(t)-F_{2n_2}(t) \right) ^2 dF_n(t). \end{aligned}$$

Let us now address the asymptotic distribution of the proposed tests. As previously discussed, the a.s. representation for $F_{jn_j}(t)- F_j(t)$ in Gijbels and Wang (1993) is given in an interval of the form $[a_{F_j},b_j]$, where $b_j < b_{W_j}$. Let $\tilde{a}_{F} = \min _j \{a_{F_j}\}$ and $b = \min _j \{b_j\}$. Note that, under $H_0$, $\tilde{a}_F = a_F$. In practice, the supremum in (7) and the integral in (10) can be computed as restricted to the interval $[\hat{a},b]$ as defined in (9), and this is how the test statistics are implemented. The theoretical results will be established for modified versions of the Kolmogorov–Smirnov and Cramér–von Mises test statistics where the supremum and the integral are restricted to an interval of the form $[\tilde{a}_F,b]$ with $b< \min _{1 \le j \le k} b_{W_j}$, since weak convergence of the involved empirical processes is jointly guaranteed only on such an interval. These modified statistics will be still denoted by $D_{KS}$ and $D_{CvM}$. For simplicity, let us denote $V_{n_j}(t) = \sqrt{n_j} \left( F_{jn_j}(t) - F_n(t) \right)$, for $j \in \{1, \ldots , k\}$. Let us consider the *k*-dimensional stochastic process

$$\begin{aligned} \boldsymbol{V_n}(t) = \left( V_{n_1}(t), \ldots , V_{n_k}(t) \right) ^\top , \end{aligned}$$

for $t \in [\tilde{a}_{F},b]$. The superscript $\top$ denotes the transpose of the corresponding vector. The asymptotic behaviour of $\boldsymbol{V_n}(t)$, which is tackled in the following theorem, will be key to obtain the asymptotic null distributions of the proposed test statistics. The proof is deferred to Appendix A.

#### Theorem 1

Let us assume that $X_1, \ldots , X_k$ are continuous random variables, $a_{G_j} < a_{F_j}$, $b_{G_j} \le b_{F_j}$ and $n_j / n \longrightarrow \pi _j \in (0,1)$, as $n_1, \ldots , n_k \longrightarrow \infty$, for all $j \in \{1, \ldots , k\}$. Let *F* be the common target distribution function under $H_0$, $a_F$ the lower endpoint of its support, and let $b < b_{W_j}$, for all $j \in \{1, \ldots , k\}$. Then, under $H_0$, $\boldsymbol{V_n}(t)$ converges weakly in $\mathbb {D}[a_F,b]^k$ to a *k*-dimensional centred Gaussian process, say $\boldsymbol{V}(t) = ( V_1(t), \ldots , V_k(t))^\top$, with covariance function given by

11 $$\begin{aligned} Cov \left( V_j(t), V_l(s) \right) = \sum _{m=1}^k p_m^2 \sqrt{\frac{\pi _j \pi _l}{ \pi _m^2}} \Gamma _m(t,s) - p_j \sqrt{\frac{\pi _l}{\pi _j}} \Gamma _j(t,s) - p_l \sqrt{\frac{\pi _j}{\pi _l}} \Gamma _l(t,s), \end{aligned}$$

if $$j \not = l$$, and

$$\begin{aligned} Cov \left( V_j(t), V_j(s) \right) = \sum _{m=1}^k p_m^2 \frac{\pi _j}{\pi _m} \Gamma _m(t,s) + (1-2 p_j) \Gamma _j(t,s), \end{aligned}$$

for $t,s \in [a_F,b]$, and where *F* is the common distribution function under the null hypothesis and $\Gamma$ is defined in (5).

An immediate application of the continuous mapping theorem yields the following result, which states the asymptotic null distribution of $D_{KS}$.

#### Corollary 1

Suppose that assumptions in Theorem 1 hold. Then, under $H_0$,

$$\begin{aligned} D_{KS} \longrightarrow \sup _{a_F \le t \le b} \sum _{j=1}^k V_j(t)^2 \end{aligned}$$

in distribution as $n_1, \ldots , n_k \longrightarrow \infty$.

The proof of the following corollary, which states the null distribution of the Cramér–von Mises-type statistic under the null hypothesis, is deferred to the Appendix A.

#### Corollary 2

Suppose that assumptions in Theorem 1 hold. Then, under $H_0$,

$$\begin{aligned} D_{CvM} \longrightarrow \sum _{j=1}^k \int _{a_F}^b V_j(t)^2 dF(t) \end{aligned}$$

in distribution as $n_1, \ldots , n_k \longrightarrow \infty$.

Although the asymptotic null distributions of the proposed test statistics are detailed in Corollary 1 and Corollary 2, they both depend on unknown population quantities. This motivates the bootstrap resampling plan proposed in Sect. 3.2 as an alternative to the theoretical results for approximating the asymptotic null distributions. To finish with this section, we deal now with the behaviour of $D_{KS}$ and $D_{CvM}$ under the alternative hypothesis, which is addressed in the following theorem.

#### Theorem 2

Let us assume that $X_1, \ldots , X_k$ are continuous random variables, $a_{G_j} < a_{F_j}$, $b_{G_j} \le b_{F_j}$ and $n_j / n \longrightarrow \pi _j \in (0,1)$, as $n_1, \ldots , n_k \longrightarrow \infty$, for all $j \in \{1, \ldots , k\}$. Then, under $H_1$, it holds that

$$\begin{aligned} D_{KS}, D_{CvM} \longrightarrow \infty , \end{aligned}$$

in probability as $n_1, \dots , n_k \longrightarrow \infty$.

The proof of the previous result is deferred to Appendix A. Theorem 2 implies that the proposed tests are omnibus, in contrast to the classical rank-based tests, which were, until now, the only statistical technique available to address the *k*-sample problem when data are subject to both left truncation and right censoring. In other words, to the best our knowledge we are indeed proposing the first omnibus tests to address the comparison of populations for left-truncated and right-censored data.

Note that the asymptotic results are derived on intervals of the form $[\tilde{a}_F,b]$ for $b < \min _j b_{W_j}$, as discussed before Theorem 1. Theorem 2 above guarantees the consistency of the proposed tests, which can detect any kind of alternatives on $[\tilde{a}_{F},b]$. Unfortunately, the limitations in the results of the convergence of $\sqrt{n_j}( F_{jn_j}(t) - F_j(t) )$, caused by the truncation and censoring, prevent the theoretical results to be obtained anywhere on the right of the smallest $b_{j}$, thus any difference among the *k* distributions in such a region may not be detected by the proposed tests. Note that this is not the case when the differences among the distributions are found on the left of any of the $a_{F_j}$. Indeed, although the convergence results in Gijbels and Wang (1993) are not explicitly stated for $t < a_{F_j}$, the process vanishes in that region, thus its behaviour is known precisely, and the consistency can be guaranteed in $[\tilde{a}_F,b]$.

### Approximation of the null distribution via bootstrap

The results in the previous section proved that the asymptotic null distributions of the proposed test statistics depend on unknown population quantities, which are to be estimated if one wanted to use the theoretical results. As an alternative, we propose a bootstrap resampling plan in order to approximate the null distribution of the test statistics and thus the *p*-value of the corresponding tests. The method below is based on the obvious bootstrap proposed in Wang (1991) and studied in detail in Gross and Lai (1996a) and Bilker and Wang (1997).

The dependence between $U_j$ and $C_j$ makes the estimation of the joint cumulative distribution function of the pair $(U_j,C_j)$ a difficult problem, and this prevents the simulation of the bootstrap truncation and censoring times from such an estimator. In order to circumvent the problem caused by this dependence, following the ideas in Wang (1991), we will consider the residual censoring variable $D_j$, which is defined by $D_j = C_j - U_j$. This variable $D_j$ represents the time from the truncation time until censoring and, naturally, it can only be observed for the censored individuals. Then, note that the censoring random variable can be written as $C_j = U_j + D_j$ and thus $Y_j = \min \{X_j, U_j+D_j\}$ and $\Delta _j = \mathbb {I} \left[ X_j \le U_j+D_j \right]$. Let $j \in \{1, \ldots , k\}$ and let $Q_j(t) = \mathbb {P} \left( D_j \le t \right)$. We will introduce the following new assumption:

12 $$\begin{aligned} D_j \text{ is } \text{ independent } \text{ of } (U_j,X_j), \text{ conditionally } \text{ on } U_j \le X_j. \end{aligned}$$

The previous assumption is natural when assuming $\mathbb {P}(U_j \le C_j) = 1$ (see Qian and Betensky 2014, for more details on assumptions with left-truncated and right-censored data). Note that the independence of $X_j$ and $(U_j,C_j)$ and assumption (12) yield the independence of $X_j-U_j$ and $D_j$ on the observable region. Under (12), the Kaplan-Meier estimator obtained from $\left( Y_{j1} - U_{j1}, 1-\Delta _{j1} \right) , \ldots , \left( Y_{jn_j} - U_{jn_j}, 1-\Delta _{jn_j} \right)$ is actually the maximum likelihood estimator of $Q_j$, say $Q_{jn_j}$ (see Wang 1991).

Let us consider the estimators of the distribution functions of the target and truncation variables, $F_{jn_j}$ and $G_{jn_j}$, as given in (2) and (3), respectively. We will first explain how to simulate a bootstrap resample under left truncation and right censoring. The following method was introduced in Wang (1991) as an extension to left-truncated and right-censored data of the obvious bootstrap proposed in Efron (1981)) for purely right-censored data. For each $j \in \{1, \ldots , k\}$,

1. Draw independently values from ${G}_{jn_j}$, ${F}_{jn_j}$ and ${Q}_{jn_j}$, say $U_{j}^{b}$, $X_{j}^{b}$ and $D_{j}^{b}$.
2. If $X_{j}^{b} \le U_{j}^{b}$, construct the triplet $(U_{j}^{b}, Y_{j}^{b}, \Delta _{j}^{b} )$, where $Y_{j}^{b} = \min \left\{ X_{j}^{b},U_{j}^{b} + D_{j}^{b} \right\}$ and $\Delta _{ji}^{b} = \mathbb {I} \left[ X_{j}^{b} \le U_{j}^{b} + D_{j}^{b} \right]$. Otherwise, dismiss the simulated values and draw again.
3. Repeat the two previous steps until completing a left-truncated, right-censored sample of size $n_j$.

The previous simulation algorithm can be employed to design the following resampling plan, which is used to approximate the null distribution of the proposed test statistics. The algorithm introduces the null hypothesis by resampling the times to event from $F_n$, which is a weighted sum of the estimators in each sample. Since the truncation and censoring variables are not assumed to be equally distributed under $H_0$, the truncation and censoring times are drawn from the estimators of the corresponding sample. Let $b \in \{1, \ldots , B\}$,

1. For each $j \in \{1, \ldots , k\}$, draw a value from the set $\{1, \ldots , k\}$ with probabilities $p_1, \ldots , p_k$, respectively, say $j^*$. Then, draw a value from ${G}_{jn_j}$, ${F}_{{j^*}n_{j^*}}$ and ${Q}_{jn_j}$, say $U_{j}^{b}$, $X_{j^*}^{b}$ and $D_{j}^{b}$. Dismiss the simulated values if $X_{j^*}^{b} > U_{j}^{b}$. Otherwise, construct the triplet $(U_{j}^{b}, Y_{j}^{b}, \Delta _{j}^{b} )$, where $Y_{j}^{b} = \min \{ X_{j^*}^{b},U_{j}^{b} + D_{j}^{b} \}$ and $\Delta _{j}^{b} = \mathbb {I} [ X_{j^*}^{b} \le U_{j}^{b} + D_{j}^{b} ]$. Repeat until completing a left-truncated, right-censored resample of size $n_j$.
2. Compute the test statistics from the *k* *b*-th bootstrap resamples, say $D_{KS}^{b}$ and $D_{CvM}^{b}$.

The result of the previous algorithm are two sets of values of the proposed statistics under the null hypothesis: one of $D_{KS}$ and one of $D_{CvM}$. Both can be used to approximate the null distribution of the corresponding test statistic. Then, since $H_0$ is rejected for large values of $D_{KS}$, we can approximate the *p*-value of the test as

$$\begin{aligned} p_{KS} = \frac{1}{B} \sum _{b=1}^{B} \mathbb {I} \left[ D_{KS}^{b} \ge D_{KS}^0 \right] , \end{aligned}$$

where $D_{KS}^0$ is the test statistic computed with the *k* original samples. So, for a chosen significance level $\alpha \in (0,1)$, the null hypothesis will be rejected by the Kolmogorov–Smirnov-type test if $p_{KS} < \alpha$. Analogously, for the Cramér–von Mises-type test, the *p*-value is approximated as

$$\begin{aligned} p_{CvM} = \frac{1}{B} \sum _{b=1}^{B} \mathbb {I} \left[ D_{CvM}^{b} \ge D_{CvM}^0 \right] , \end{aligned}$$

where $D_{CvM}^0$ is the Cramér–von Mises statistic computed from the *k* original samples. Similarly, the null hypothesis will be rejected by the Cramér–von Mises-type test if $p_{CvM} < \alpha$.

Note that, in the proposed bootstrap algorithm, one first has to determine which sample the times to event are drawn from, due to the weighted-average structure of $F_n$. However, the truncation and residual censoring times are always drawn from the distribution function estimator of the corresponding sample.

Alternatively, one could try to use the simple bootstrap, described in Gross and Lai (1996a) and Bilker and Wang (1997), to approximate the null distribution of the proposed test statistics. This method builds bootstrap resamples by sampling from the original triplets with uniform probabilities $1/n_j$, for each $j \in \{1, \ldots , k\}$. Although consistent (Gross and Lai 1996a) and computationally faster than the obvious bootstrap, this method does not take the internal structure of the data into account, as stated in the two aforementioned references. Not only that, but it is not possible to use it straightforwardly to design a bootstrap resampling plan to approximate the null distributions of the proposed statistics, since one must introduce the null hypothesis while taking into account the different truncation and censoring distributions across the *k* groups. Thus, the application of the simple bootstrap in our context is unclear.

Besides the simulation results in Sect. 4, which will demonstrate the good performance of the previous algorithm to approximate the null distribution of the proposed test statistics, Theorem 1 in Bilker and Wang (1997) and Theorem 1 above allow to prove the adequacy of the method theoretically. Before stating the results, it is necessary to introduce new notation. For $j \in \{1, \ldots , k\}$, let $F^{b}_{jn_j}$ be the bootstrap estimator of the distribution function of the target variable. Let $D^{b}_{KS}$ and $D^{b}_{CvM}$ be the proposed tests computed using the bootstrap resamples. The object of study is now the process

$$\begin{aligned} \boldsymbol{V^{b}_{n}}(t) = (V^{b}_{n_1}(t), \ldots , V^{b}_{n_k}(t))^\top , \end{aligned}$$

where $$V^{b}_{n_j}(t) = \sqrt{n_j} \left( F^{b}_{jn_j}(t) - F^{b}_n(t) \right) $$ and $$F_n^b(t) = \sum _{j=1}^k p_j F_{jn_j}^b(t)$$.

#### Theorem 3

Suppose that the assumptions in Theorem 1 and that condition (12) hold. Then, under $H_0$, $\boldsymbol{V^{b}_{n}}(t)$ converges weakly, conditionally on the original data, in $\mathbb {D}[a_F,b]^k$ to a *k*-dimensional centred Gaussian process, say $\boldsymbol{V^{b}}(t) = ( V^{b}_1(t), \ldots , V^{b}_k(t))^\top$, with the covariance function given in (11).

The proof of the previous result is deferred to Appendix A. It follows the lines of the proof of Theorem 1 above and uses Theorem 1 in Bilker and Wang (1997) and the fact that $F^{b}_{jn_j}(t) \longrightarrow F_{jn_j}(t)$ a.s. under $H_0$. This has not been stated explicitly in Bilker and Wang (1997), but it is an immediate consequence of the asymptotic representation obtained in the Lemma 3 of that paper, where the continuity of $F_j$ plays a fundamental role. Theorem 3 allows to derive the following two asymptotic distributions.

#### Corollary 3

Suppose that the assumptions in Theorem 1 and that condition (12) hold. Then

$$\begin{aligned} D^{b}_{KS} \longrightarrow \sup _{a_F \le t \le b} \sum _{j=1}^k V_j(t)^2 \end{aligned}$$

in distribution as $n_1, \ldots , n_k \longrightarrow \infty$, conditionally on the original data, where $V_j(t)$ are the limit processes in Theorem 1.

#### Corollary 4

Suppose that the assumptions in Theorem 1 and that condition (12) hold. Then

$$\begin{aligned} D^{b}_{CvM} \longrightarrow \sum _{j=1}^k \int _{a_F}^b V_j(t)^2 dF(t) \end{aligned}$$

in distribution as $n_1, \ldots , n_k \longrightarrow \infty$, conditionally on the original data, where $V_j(t)$, for $j \in \{1, \ldots , k\}$, are the limit processes in Theorem 1.

By means of Corollary 1 and Corollary 3, $D_{KS}$ and $D^{b}_{KS}$ have the same asymptotic distribution, which justifies the validity of the proposed bootstrap resampling plan. An analogous statement can be claimed for $D_{CvM}$ and $D_{CvM}^{b}$ following Corollary 2 and Corollary 4.

## Simulation study

The practical implementation of the methods described above is not straightforward, since the truncation causes problems when the risk set $r_{ji}$ is unitary, as reported by Lai and Ying (1991) and Gross and Lai (1996a). More in particular, we will focus on the so-called *holes*, which are times to event with corresponding unitary risk sets. Of course, the largest time to event is always a hole, but it will not be considered in the following discussion. Due to the product-like formula of the estimator of the survival function, a unitary risk set causes the estimator to be zero from the hole on, which may lead to an inconsistent estimator, so that the sooner the hole appears, the more harmful it is for estimation purposes. Since many methods depend on the estimation of the survival function, holes affect in many ways. In particular for our interests, even though $H_0$ is true, a hole can cause a distance between two estimators to be big enough so that the test rejects the null hypothesis. Moreover, regarding the resampling plan in Sect. 3.2, if there is a hole in the sample, the estimator would not assign mass to various (possibly many) observed times to event, so that they will not appear in the resamples either, which could lead to defective bootstrap resamples. Needless to say, holes appear with purely left-truncated data as well (see Woodroofe 1985, and Strzałkowska-Kominiak and Stute 2010, for theoretical results and simulations on this matter). Precisely, in these two mentioned papers, it is shown that the probability of a hole to appear in a truncated sample tends to zero as the sample size grows. In other words, the problem of holes only affects to small samples. Despite this result being proved for purely right-truncated data, it is immediate to see that it also holds under left truncation. Moreover, when data are subject to both left truncation and right censoring, taking into account that the observed variable *Y* is left-truncated by *U* and following the definition of the risk set $r_{ji}$, the previous results can be extended to the current setting and hence the problem of holes is restricted to small samples.

In order to circumvent the problem of holes, different solutions have been proposed in the literature. The estimation of the survival function conditioned to times to event bigger than the greatest hole is suggested in Tsai et al. (1987) and Klein and Moeschberger (2003). However, this method results in the estimation of a function different from the one initially of interest. Alternatively, following the idea in Stute and Wang (2008) for left-truncated data, we propose to employ the following modified distribution function estimator

13 $$\begin{aligned} \widehat{F}_{jn_j}(t) = 1 - \prod _{x_{ji} \le t} \left( 1 - \frac{d_{ji}}{r_{ji}+1} \right) . \end{aligned}$$

That is, increasing the risk set in 1 unity in order to avoid the value of 1 in the denominator above. The estimator (13) was implemented into R (R Core Team 2024). There is, however, software to compute the estimation with left-truncated and right-censored data, although the previous correction in the risk sets is not incorporated anywhere to the best of our knowledge. For example, the function survfit of the package survival (Therneau 2026) can be employed. Nevertheless, there are some remarks that one should take into consideration before employing such a function. On the one hand, the risk sets are defined as $\left\{ (U_{jl}, Y_{jl}) \mid U_{jl}< x_{ji} < Y_{jl} \right\}$. That is, for an individual to be observed at time $x_{ji}$, the truncation time has to be strictly smaller that $x_{ji}$. This is not the case for the risk sets in either (2) or (13). This is not relevant when data are simulated from continuous distributions, but it is important when ties are present in the sample, since there could exist pairs $(U_{jl}, Y_{jl})$ such that $U_{jl} = x_{ji}$, for some observed time to event $x_{ji}$. Related to that, the function survfit in the package survival does not allow one to use pairs of data such that $U_{ji} = Y_{ji}$.

The algorithm proposed in Giacomini et al. (2013) to speed up computation times in Monte Carlo studies was employed. It basically allows to work with just one bootstrap resample in each Monte Carlo trial. As a consequence, the number of bootstrap statistics equals the number of Monte Carlo trials. The result of the algorithm are *M* statistics (one for each Monte Carlo trial) and *M* bootstrap statistics under $H_0$, which are used to approximate the null distribution of the test statistic. The *p*-value for the test in each of the *M* Monte Carlo trials is then approximated as the proportion of the *M* statistics under $H_0$ that are larger or equal to it. The previous method is guaranteed to work for large values of *M* and large sample sizes.

Let us now study the bootstrap procedure described in the Sect. 3.2. Table 2 contains rejection proportions for different simulation scenarios, described in Table 1, where the nontruncation probabilities $\gamma _{T_j} = \mathbb {P}(U_j \le X_j)$ and noncensoring probabilities, namely $\gamma _{\Delta _j}=\mathbb {P}(X_j \le C_j \mid U_j \le X_j)$, are also reported. Note that W(a,b) refers to the Weibull distribution with shape parameter a and scale parameter b; $N(\mu ,\sigma ^2)$ denotes the Normal distribution with mean $\mu$ and variance $\sigma ^2$; and Exp($\lambda$) represents the Exponential distribution with mean $1/\lambda$. Also note that balanced and unbalanced samples sizes are considered and that all scenarios are under the null hypothesis. Since the target variable is the same in the three populations, the empirical rejection probabilities should be close to $\alpha =0.05$. This indeed happens for every combination of sizes, except maybe for (50, 100, 150), where the proportion of rejections is slightly below the nominal level in some scenarios. However, as the sample sizes increase, the rejection proportions approach the chosen nominal level. This indicates that the proposed bootstrap procedure leads to a correctly calibrated test.

**Table 1:** Simulation scenarios under $H_0$, with their corresponding nontruncation and noncensoring probabilities

		$X_j$	$U_j$	$D_j$	$\gamma _{T_j}$	$\gamma _{\Delta _j}$
Scenario 1	Group 1	N(4,1)	N(3,1)	Exp(1)	0.76	0.48
	Group 2	N(4,1)	N(3,1)	Exp(1)	0.76	0.48
	Group 3	N(4,1)	N(3,1)	Exp(1)	0.76	0.48
Scenario 2	Group 1	N(4,1)	N(3,1)	Exp($\frac{1}{2}$)	0.76	0.63
	Group 2	N(4,1)	N(3,1)	Exp(1)	0.76	0.48
	Group 3	N(4,1)	N(3,1)	Exp(2)	0.76	0.37
Scenario 3	Group 1	N(4,1)	N(3,1)	Exp($\frac{1}{2}$)	0.76	0.63
	Group 2	N(4,1)	N(3,1)	Exp(1)	0.76	0.48
	Group 3	N(4,1)	N(4,1)	Exp(2)	0.50	0.63
Scenario 4	Group 1	Exp($\frac{1}{2}$)	Exp($\frac{1}{2}$)	Exp(2)	0.50	0.60
	Group 2	Exp($\frac{1}{2}$)	Exp($\frac{1}{2}$)	Exp(1)	0.50	0.67
	Group 3	Exp($\frac{1}{2}$)	Exp(1)	Exp(1)	0.67	0.56
Scenario 5	Group 1	W(4,3)	N(1,2)	Exp($\frac{1}{4}$)	0.79	0.82
	Group 2	W(4,3)	N(2,2)	Exp($\frac{1}{2}$)	0.63	0.88
	Group 3	W(4,3)	W(2,3)	Exp(1)	0.55	0.70
Scenario 6	Group 1	W(4,3)	N(2,$\frac{1}{2}$)	Exp($\frac{1}{4}$)	0.78	0.97
	Group 2	W(4,3)	N(2,3)	Exp(1)	0.59	0.70
	Group 3	W(4,3)	Exp(1)	Exp($\frac{1}{4}$)	0.91	0.98

**Table 2:** Rejection proportions for samples of sizes $(n_1,n_2,n_3)$ from Table 1, $\alpha =0.05$ and $M=5000$ Monte Carlo replications

	(100, 100, 100)		(50, 100, 150)		(200, 200, 200)		(100, 200, 300)		(500, 500, 500)		(250, 500, 750)	
	KS	CvM	KS	CvM	KS	CvM	KS	CvM	KS	CvM	KS	CvM
Scenario 1	0.0408	0.0492	0.0444	0.0396	0.0596	0.0496	0.0524	0.0480	0.0502	0.0480	0.0460	0.0502
Scenario 2	0.0602	0.0398	0.0352	0.0348	0.0570	0.0428	0.0386	0.0472	0.0524	0.0468	0.0426	0.0484
Scenario 3	0.0448	0.0478	0.0456	0.0486	0.0540	0.0510	0.0544	0.0464	0.0532	0.0498	0.0526	0.0532
Scenario 4	0.0472	0.0496	0.0458	0.0524	0.0442	0.0530	0.0430	0.0516	0.0484	0.0562	0.0530	0.0490
Scenario 5	0.0528	0.0400	0.0362	0.0370	0.0520	0.0488	0.0482	0.0550	0.0516	0.0482	0.0494	0.0470
Scenario 6	0.0444	0.0470	0.0322	0.0338	0.0518	0.0490	0.0466	0.0532	0.0496	0.0462	0.0558	0.0528

Let us now address the power of the proposed tests by means of the results in Table 4. There are five simulation scenarios with three populations in each of them, where one of the target variables is different from the other two in the first five scenarios, and three different distributions in the last one. In addition to the proposed tests, weighted rank-based tests will be considered for comparison. The test statistics are based on the quantity

$$\begin{aligned} L_j = \sum _{i=1}^D W(t_i) \left( d_{ji} - d_i \frac{r_{ji}}{r_i}\right) , \end{aligned}$$

where $t_1, \ldots , t_D$ are the distinct observed event times in the *k* combined samples, $d_i =\sum _{j=1}^k d_{ji}$ is the number of events taking place at time $t_i$ in the *k* combined samples, $r_i =\sum _{j=1}^k r_{ji}$ is the total number of individuals at risk at time $t_i$ in the *k* combined samples, and $W(\cdot )$ is a weight function (see Klein and Moeschberger 2003, for more details on rank-based tests). We will consider weights of the form

$$\begin{aligned} W(t_i)= \tilde{S}_{n}(t_{i-1})^p \tilde{F}_{n}(t_{i-1})^q, \text{ with } p,q \ge 0, \end{aligned}$$

where $\tilde{S}_{n}$ is the survival function estimator of the *k* combined samples and $\tilde{F}_{n} = 1 - \tilde{S}_{n}$. Note that, if $p=q=0$, one obtains the expression of the classical log-rank test (LR) (Mantel 1966; Peto and Peto 1972) adapted to left-truncated and right-censored data. In order to interpret the simulation results, it is important to know that the log-rank test is optimal under proportional hazards (see Klein and Moeschberger 2003, for more details). If $p=1$ and $q=0$, the test assigns more weight to early event times, so the resulting test, which will be denoted by LR$^{\text{ E }}$, will be more sensitive to departures of the null in the left part of the support of the target variables under consideration. On the other hand, if $p=0$ and $q=1$, the resulting test, denoted by LR$^{\text{ L }}$, will be better to detect differences in the right tails of the distributions. Finally, we will also consider a test that targets to differences in the central part of the support of the distributions, which is obtained by setting the weights $p=q=0.5$, and will be denoted by LR$^{\text{ M }}$.

In Scenario 7 of Table 4, the three target variables follow an exponential distribution, so it is a scenario under proportional hazards. The rejection proportions of the rank-based tests are clearly higher than those of the proposed tests in this Scenario 7, which could have been anticipated. However, $D_{KS}$ and $D_{CvM}$ are powerful for large sample sizes as well. The rank-based tests perform better than the proposed tests in Scenario 8 as well, being LR$^{\text{ E }}$, the most powerful among the five tests. The better performance of LR could be explained because the target variables are normally distributed and differ only in the mean parameter, thus are stochastically ordered, so that the survival functions do not cross. However, $D_{KS}$ and $D_{CvM}$ are powerful for large sample sizes as well. In Scenario 9, where the times to event follow Weibull distributions, the proposed tests clearly outperform the log-rank test, whose rejection proportions are low, even for big sample sizes. However, LR$^{\text{ L }}$ is the most powerful test in this scenario. In Scenario 10, the times to event follow Weibull distributions as well, however, the parameters are set to reveal the weakness of the log-rank test. Indeed, the rejection proportions of LR are close to the significance level, no matter the sample sizes, which implies that it fails to reject the null hypothesis. This is not surprising, as the log-rank test is not omnibus. This is in contrast with the proposed tests, which perform well and, again, their rejection proportions are close to 1 with large sample sizes. In this case, both LR$^{\text{ E }}$ and LR$^{\text{ L }}$ outperform the log-rank test, but the rejection proportions are still far from those of $D_{KS}$ and $D_{CvM}$. Scenario 11 is intended to illustrate the strength of the Kolmogorov–Smirnov-type test, since there is a large vertical gap between the distribution functions. The rejection proportions reveal that $D_{KS}$ is indeed the most powerful test in this case, the Cramér–von Mises test performs fairly well and the rank-based tests have trivial power, as the rejection proportions are also close to the significance level for the three different sample sizes. In Scenarios 12 and 13, we considered different target distributions from different parametric families. In the former, LR$^{\text{ M }}$ turns out to be the most powerful test, but the proposed tests exhibit large power when $n_j=500$ $j \in \{1,2,3\}$; as in the latter, the proposed $D_{KS}$ and $D_{CvM}$ are more powerful than the rank-based tests. It is also interesting to see how the proposed tests perform well in both cases, despite the quite high censoring probabilities.

**Table 3:** Simulation scenarios under $H_1$, with their corresponding nontruncation and noncensoring probabilities

		$X_j$	$U_j$	$D_j$	$\gamma _{T_j}$	$\gamma _{\Delta _j}$
Scenario 7	Group 1	Exp(1)	Exp(2)	Exp$\left( \frac{1}{2} \right)$	0.67	0.78
	Group 2	Exp(1)	Exp(4)	Exp(1)	0.80	0.60
	Group 3	Exp$\left( \frac{1}{2} \right)$	Exp(1)	Exp(1)	0.67	0.55
Scenario 8	Group 1	N(4,1)	N(3,1)	Exp$\left( \frac{1}{2} \right)$	0.75	0.63
	Group 2	N(4,1)	N(4,1)	Exp(1)	0.50	0.71
	Group 3	N(4.5,1)	N(4,1)	Exp$\left( \frac{1}{2} \right)$	0.64	0.73
Scenario 9	Group 1	W(4,3)	N(1,1)	Exp$\left( \frac{1}{4} \right)$	0.91	0.67
	Group 2	W(4,3)	W(2,2)	Exp(1)	0.79	0.48
	Group 3	W(2,3)	N(1,1)	Exp$\left( \frac{1}{2} \right)$	0.68	0.63
Scenario 10	Group 1	W(4,2)	Exp($\frac{1}{2}$)	Exp$\left( \frac{1}{4} \right)$	0.58	0.86
	Group 2	W(4,2)	Exp($\frac{1}{2}$)	Exp$\left( \frac{1}{4} \right)$	0.58	0.86
	Group 3	W(3,1.9)	Exp($\frac{1}{2}$)	Exp$\left( \frac{1}{4} \right)$	0.55	0.88
Scenario 11	Group 1	U(0,2)	U(0,2)	U(0,1)	0.50	0.71
	Group 2	U(0,2)	U(0,1)	U(0,2)	0.75	0.71
	Group 3	U(1,2)	U(1,2)	U(0,1)	0.50	0.83
Scenario 12	Group 1	W(6,3)	W(5,3)	Exp(2)	0.51	0.37
	Group 2	W(4,3)	W(4,3)	Exp(1)	0.50	0.50
	Group 3	N(3,1)	N(3,1)	Exp(1)	0.50	0.43
Scenario 13	Group 1	W(2,4)	W(5,3)	Exp(2)	0.62	0.19
	Group 2	U(0,6)	U(0,4)	U(0,2)	0.67	0.25
	Group 3	N(4,1)	N(3,1)	Exp(1)	0.76	0.32
Scenario 14	Group 1	E$_1$	U(0,8)	Exp(2)	0.57	0.31
	Group 2	E$_2$	U(0,8)	Exp(2)	0.57	0.31
	Group 3	E$_2$	U(0,8)	Exp(2)	0.57	0.31
Scenario 15	Group 1	Exp(1/10)	U(0,10)	Exp(3)	0.63	0.23
	Group 2	L$_2$	U(0,10)	Exp(3)	0.63	0.29
	Group 3	L$_2$	U(0,10)	Exp(3)	0.63	0.29

**Table 4:** Rejection proportions for $\alpha =0.05$, $M=5000$ Monte Carlo replications and three different sample sizes for the proposed Kolmogorov–Smirnov (KS) and Cramér–von Mises (CvM) tests, along with the log-rank test (LR) and weighted rank test which target to detect early (LR$^{\text{ E }}$), mid (LR$^{\text{ M }}$) and late (LR$^{\text{ L }}$) differences

	$n_j=100$						$n_j=200$						$n_j=500$					
	KS	CvM	LR	LR$^{\text{ E }}$	LR$^{\text{ M }}$	LR$^{\text{ L }}$	KS	CvM	LR	LR$^{\text{ E }}$	LR$^{\text{ M }}$	LR$^{\text{ L }}$	KS	CvM	LR	LR$^{\text{ E }}$	LR$^{\text{ M }}$	LR$^{\text{ L }}$
Scenario 7	0.3256	0.4816	0.8948	0.8082	0.8832	0.8560	0.6016	0.7982	0.9970	0.9982	0.9962	0.9932	0.9744	0.9964	1	1	1	1
Scenario 8	0.2224	0.2832	0.3246	0.5202	0.4146	0.3908	0.3862	0.5316	0.6554	0.8364	0.7236	0.6530	0.8004	0.9020	0.9784	0.9940	0.9278	0.9684
Scenario 9	0.1834	0.2404	0.0834	0.0984	0.2374	0.6594	0.6438	0.7294	0.1714	0.1438	0.4316	0.9392	0.9960	0.9996	0.5138	0.3036	0.8214	1
Scenario 10	0.2512	0.2566	0.0594	0.1984	0.0532	0.1308	0.5638	0.5730	0.0534	0.3416	0.0572	0.2044	0.9398	0.9418	0.0488	0.6964	0.0504	0.4606
Scenario 11	0.6070	0.3192	0.0538	0.0592	0.0510	0.0548	0.8862	0.6128	0.0560	0.0512	0.0532	0.0588	0.9994	0.9352	0.0514	0.0484	0.0490	0.0526
Scenario 12	0.0646	0.2148	0.0926	0.1362	0.3622	0.1954	0.3408	0.5538	0.1466	0.2608	0.6820	0.3520	0.9376	0.9818	0.2822	0.6016	0.9980	0.7114
Scenario 13	0.9544	0.2018	0.0558	0.0922	0.0868	0.0980	0.9962	0.5756	0.0686	0.1548	0.1096	0.1592	1.0000	0.9530	0.0980	0.3948	0.2044	0.3408
Scenario 14	0.2682	0.1666	0.0554	0.0898	0.0690	0.0686	0.5760	0.3706	0.0550	0.1136	0.0810	0.0830	0.9560	0.8582	0.0616	0.1934	0.1216	0.1248
Scenario 15	0.0734	0.1282	0.3636	0.0530	0.3356	0.8708	0.1730	0.3114	0.6750	0.0548	0.6228	0.9964	0.3310	0.6012	0.9824	0.0568	0.9448	1.0000

**Fig. 1:**
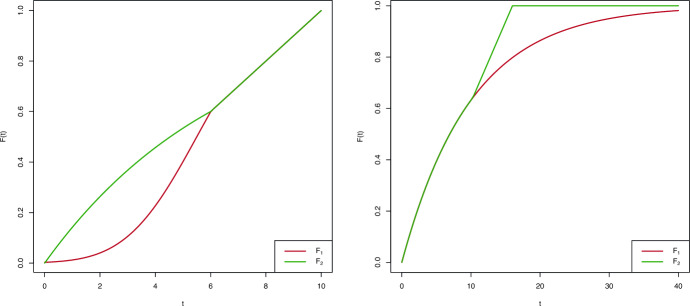
Target variables of Scenario 14 (left) and Scenario 15 (right) in Table 3

In order to get a deeper understanding of the performance of the tests, we also considered one scenario with early differences and one with late differences among the three distribution functions. In Scenario 14 in Table 3, the target variable in the first group, denoted by $E_1$, is as follows:

$$\begin{aligned} E_1 = Z_{1E} \mathbb {I}[Z_{1E} \le 6] + U_{1E} \mathbb {I}[Z_{1E} > 6], \end{aligned}$$

where $Z_{1E}$ and $U_{1E}$ are independent random variables such that $Z_{1E} \sim \text{ N }(5.5,2)$ and $U_{1E} \sim \text{ U }(6,10)$. The distribution function of $E_1$ is depicted as the red curve in the left panel of Fig. 1. Similarly, for the distribution in the second and third groups, denoted by $E_2$, is

$$\begin{aligned} E_2 = Z_{2E} \mathbb {I}[Z_{2E} \le 6] + U_{2E} \mathbb {I}[Z_{2E} > 6], \end{aligned}$$

where $Z_{2E}$ and $U_{2E}$ are independent random variables such that $Z_{2E} \sim \text{ Exp }(0.1527)$ and $U_{2E} \sim U(6,10)$. The distribution function of $E_2$, say $F_2$ is depicted as the green curve in the left panel of Fig. 1. We considered the same truncation variables in the three groups, which follow a U(0,8) and, similarly the residual censoring variables follow an Exp(1/2) in the three populations. The nontruncation and noncensoring probabilities are detailed in Table 3. In Table 4, we see that the rejection proportions of the proposed tests are larger than those of the weighted rank tests, despite the high censoring probabilities. Interestingly, the LR$^{\text{ E }}$ does not perform well and also the log-rank test has trivial power.

In Scenario 15 in Table 3, the target variables in the second and third groups, denoted as $L_2$, are as follows:

$$\begin{aligned} L_2 = Z_{2L} \mathbb {I}[Z_{2L} \le 10] + U_{2L} \mathbb {I}[Z_{2L} > 10], \end{aligned}$$

where $Z_{2L}$ and $U_{2L}$ are independent random variables such that $Z_{2L} \sim \text{ Exp }(1/10)$ and $U_{2L} \sim \text{ U }(10,16)$. The distribution function of $L_2$, say $F_2$ is depicted as the green curve in the right panel of Fig. 1. The target variable in the first group follows an Exp(1/10) (red curve of the aforementioned representation). We considered the same truncation variables in the three groups, which follow a U(0,8) and, similarly the residual censoring variables follow an Exp(1/2) in the three populations. Table 4 contains the rejection proportions for the comparison of the distributions obtained from the proposed tests and three different rank-based tests. In this case, LR$^{\text{ L }}$ is the best out of the five tests, as one could expect beforehand due to the late differences among the three distributions, although LR and LR$^{\text{ M }}$ perform well too. The proposed CvM test is moderately powerful, but the KS test finds it more difficult to distinguish among the distribution functions.

Results in Table 4 reveal that the proposed tests exhibit good practical performance under all of the considered scenarios. Not only that, the new tests proved to be more versatile when detecting the alternative hypothesis than the rank-based tests, whose performance is questionable in some of the scenarios above. This better performance of the proposed tests compared to the rank-based tests in a broad range of alternatives is natural considering the omnibus nature of the proposed tests and the relation of the log-rank test and the Cox proportional hazards model. Indeed, it is well-known that the log-rank test can be derived from the Cox proportional hazards model as a test to assess the significance of the regression coefficients when dealing with a binary covariate that indicates the group of origin. In other words, this piece of work reveals that these new tests are generally preferred over a possible regression-based approach to this *k*-sample problem when no information about the alternative hypothesis is available.

Finally, $D_{CvM}$ generally outperforms $D_{KS}$ in the considered scenarios, which indicates that the Cramér–von Mises is, broadly speaking, more powerful than the Kolmogorov–Smirnov test. This is in line with the conclusions in previous papers on the comparisons of distributions, such as Schumacher (1984), where both tests are considered for the two-sample problem under right censoring; and Zhang and Wu (2007), where the *k*-sample problem with complete data is addressed. This conclusion is not restrictive to the two or *k*-sample problem, as it has also been reported in the goodness-of-fit framework, for instance in Stephens (1974).

So far, the new tests have been implemented by choosing $p_j=n_j/n$ in the construction on $F_n$ (as we stated in Sect. 3). Other choices are also possible. We have briefly explored the possibility of choosing $p_j=1/k$ for $j \in \{1,2,3\}$. We fixed $n_1 + n_2 + n_3 = 600$, and studied the power when the diverging target variable, always in group 3, has the smallest sample size ($n_3=100$), medium sample size ($n_3=200$) or the largest sample size ($n_3=300$). Table 5 contains the rejection proportions for the weights $p_j=n_j/n$ and $p_j=1/k$. We have observed that in neither of these two scenarios the choice of the weights has much impact on the power of the proposed tests.

**Table 5:** Power results for scenarios under $H_1$ from Table 3, and choices of the weights in $F_n$ as $p_j=1/k$ for all $j \in \{1,2,3\}$, and $p_j=n_j/n$

		(300, 200, 100)		(300, 100, 200)		(100, 200, 300)	
	$p_j$	KS	CvM	KS	CvM	KS	CvM
Scenario 7	1/*k*	0.4478	0.5612	0.4994	0.7698	0.6980	0.8738
	$n_j/n$	0.5514	0.5608	0.6756	0.7578	0.4850	0.8648
Scenario 8	1/*k*	0.2952	0.2630	0.4018	0.4548	0.4376	0.4962
	$n_j/n$	0.2438	0.2094	0.4406	0.4692	0.4628	0.5118

## Real data illustration

The performance of the proposed tests will be exemplified with a real dataset regarding unemployment times (see de Uña-Álvarez and Iglesias-Pérez 2010). These data are part of a survey, the so-called *Encuesta de Población Activa* (Labour Force Survey), made to Spanish unemployed people by the Spanish National Institute of Statistics. More precisely, we will focus on data regarding unemployed married women from Galicia, a region on the North-West of Spain. Data were collected in the following way: unemployed women actively looking for a job were recruited into the study and asked different questions about personal and professional life. They were then interviewed again every three months, to see whether their employment status had changed or not; that is, if they had found a job or had withdrawn the job search. The way data were collected is by cross-sectional sampling and it leads to late entry times; that is, a woman can find a job or stop the searching before being recruited and, as a consequence, she will not be included in the study. This yields undersampling of short unemployment times and left truncation.

Let us consider the total time, in months, it took the woman to find employment, which will be our target variable, $X_j$. The left truncation variable, say $U_j$, will be defined as the time, in months, that women had spent searching for a job when they were first interviewed. It is clear that only women such that $U_j \le X_j$ will be included in the study. Right censoring is also present due to administrative censoring or because individuals withdraw from the active search for employment. We acknowledge that this latter event constitutes a competing risk with respect to the event of interest, and it may induce dependence between censoring and survival times. Consequently, the assumption of independent censoring might be violated in this context. Nonetheless, we will ignore this potential issue in our illustrative example. All in all, it is clear that we are dealing with left-truncated and right-censored data.

The number of children and several other variables in this dataset were recorded at the time the women were first interviewed. For this example, three groups are going to be considered: women without children, which corresponds to $j=1$; mothers of a single child, $j=2$; and mothers of two or more children, $j=3$. The corresponding sample sizes are $n_1=246$, $n_2=413$ and $n_3=350$. By using the estimator in Wang (1991), the estimated nontruncation probabilities are $\gamma _{1n_1}=0.4521$, $\gamma _{2n_2}=0.4725$ and $\gamma _{3n_3}=0.5090$, which means that the truncation reduces all of the subsamples by half, so the loss of effective sample size is considerable. Lastly, around $80\%$ of the women are censored in each group, so the censoring probabilities are quite high as well. More in detail, only $28.7\%$ of the censored women dropped out the job search, so $71.3\%$ of them reached the end of study (18 months) without finding a job, so most of the censoring was administrative.

To apply the Kolmogorov–Smirnov and Cramér–von Mises tests proposed in this manuscript, the independence between the truncation and target variables needs to be assessed. As data in the sample are subject to the restriction $U \le X$, we are only able to test for independence in the observable region, namely test for the so-called quasi-independence. One should note that this quasi-independence assumption is sufficient for the proposed methods to hold, as reported in Tsai (1990). The package tranSurv (Chiou and Qian 2025) contains the quasi-independence tests proposed in Martin and Betensky (2005) and Austin and Betensky (2014). For this dataset, there is strong evidence against the independence in the group of women with no children, as these aforementioned tests reject the null hypothesis for 1% significance level. In contrast, such evidence was not found against quasi-independence in the group of women with a single child, nor in the group of mothers of two or more children. Taking these results into account, it is legitimate to apply the proposed methods to compare groups 2 and 3, whereas including the group of women without children looks questionable. Since this is merely an illustrative example, we will address both comparisons, but the results of the latter have to be interpreted cautiously.

**Fig. 2:**
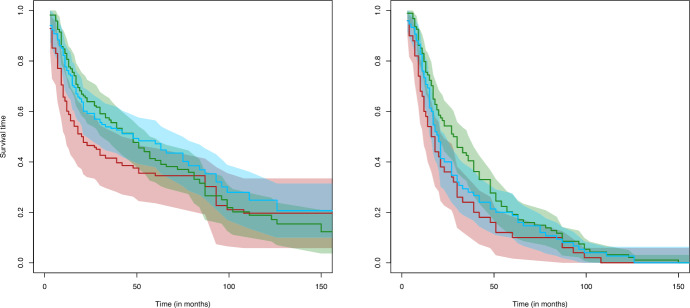
Estimated survival curves of the time looking for a job in the group of women without children, in red; with one child, in green; and mothers of two or more, in blue. In addition, pointwise confidence intervals for the three curves are depicted in each panel. The left panel contains the survival function estimators and the right one, the ordinary empirical survival functions that ignore censoring and truncation

Consider the group $j=2$ and $j=3$, namely, women with one child and mothers of two or more children. Prior to applying the tests, we wondered whether the proportional hazards assumption holds for this dataset, as this is relevant to better interpret the results, especially regarding the log-rank test. The test we performed, which accounts for left truncation and right censoring, is based on the Schoenfeld residuals of the Cox proportional hazards regression model and it is implemented in the function cox.zph of the package survival (see Chapter 6 in Therneau and Grambsch 2000, for more details). The *p*-value was 0.17, which does not reject the null hypothesis of proportional hazards among the two groups. As a consequence, we know that the log-rank test is optimal in this case. The estimated *p*-values of the proposed Kolmogorov–Smirnov and Cramér–von Mises-type tests, obtained with $B=5000$ bootstrap replications each, are 0.2032 and 0.7200, respectively. Moreover, the approximated *p*-value of the tests LR, LR$^{\text{ E }}$, LR$^{\text{ M }}$ and LR$^{\text{ L }}$ adapted to left-truncated and right-censored data are 0.42, 0.71, 0.18 and 0.28, respectively. That is, neither the proposed tests nor the rank-based tests reject the null hypothesis. Therefore, the *p*-values of the proposed tests lead to the same conclusion as the one of the log-rank test. The results are supported by the survival curves depicted in the left panel of Fig. 2, where the green and blue curves are close to each other.

Consider now the three groups introduced above. Recall that, as discussed above, the quasi-independence assumption is violated in the group of women with no children, so the results must be interpreted cautiously. In this case, the test based on the Schoenfeld residuals yields a *p*-value of 0.002, which rejects the null hypothesis of proportional hazards in the three groups, in contrast to the case where only two groups were considered. Thus, the log-rank test might not be optimal in this case. The estimated *p*-values of the proposed Kolmogorov–Smirnov and Cramér–von Mises-type tests, obtained with $B=5000$ bootstrap replications each, are 0.0420 and 0.0232, respectively. Moreover, the approximated *p*-value of the tests LR, LR$^{\text{ E }}$, LR$^{\text{ M }}$ and LR$^{\text{ L }}$ adapted to left-truncated and right-censored data are 0.76, 0.37, 0.54 and 0.19, respectively. That is, the proposed Kolmogorov–Smirnov and Cramér–von Mises-type tests reject the null hypothesis, whereas the rank-based tests do not. Therefore, the *p*-values of the proposed tests lead to different conclusions than the one of the log-rank test. The small *p*-values of $D_{KS}$ and $D_{CvM}$ are supported by the left panel in Figure $2$, where the estimated survival curves are clearly different. The largest departure among the three curves seems to be in the left tail of the survival functions, although the three pointwise confidence bands overlap at any time point.

As a remark, for this example with three groups, we have also applied the ordinary Kolmogorov–Smirnov and Cramér–von Mises tests to the observed times to event, that is, we disregard truncation and censoring. The obtained *p*-values are 0.1550 and 0.0538, respectively. In other words, the conclusion of the test changes if the truncation and censoring are not taken into consideration, which illustrates that ignoring them could lead to misleading results. The ordinary empirical survival curves are represented in the right panel of Fig. 2. In addition, in contrast to the case where truncation and censoring are taken into account, the survival function in the group of women without children falls in the confidence interval of the other two, except maybe around 50 weeks, which could explain the difference in *p*-values.

## Main conclusions

In this paper, two different tests have been proposed to address the *k*-sample problem with left-truncated and right-censored data: they are both adaptations of the Kolmogorov–Smirnov and Cramér–von Mises tests proposed in Kiefer (1959) for the *k*-sample problem with complete data. By using the fundamental results in Gijbels and Wang (1993), the asymptotic distributions of the proposed tests were derived. The behaviour of the tests under the alternative hypothesis was also investigated, which implies the consistency of both tests. The dependence of the asymptotic null distribution on unknown population quantities led to propose a bootstrap resampling plan to approximate the *p*-values of the proposed tests. Theoretical results were also derived for the bootstrap versions of the proposed statistics, which proved the consistency of the method. Moreover, a simulation study was conducted to assess the quality of the bootstrap algorithm. Simulations under the null hypothesis suggest that the proposed methods lead to a correctly calibrated test. A simulation study was also conducted to address the power with finite sample sizes of the new tests, which were also compared to the classical log-rank test and three other weighted rank-based tests. Different types of alternatives were considered, such as proportional hazards, stochastic ordered distributions, and early and late differences. The proposed tests exhibited power in every scenario, and outperformed the rank-rank based tests in many of them. Broadly speaking, the Cramér–von Mises test seems to be more powerful than the Kolmogorov–Smirnov test.

As a possible future research topic, it would be interesting to study *post-hoc* tests, which are to be used when the null hypothesis is rejected, in order to detect which pairs of distributions are different and which ones can be considered to be equal. The extension to the regression framework could also be explored, in order to incorporate information provided by multiple covariates.

## Appendix: Proofs

### Proof of Theorem 1

The proof is fundamentally based on the weak convergence results derived in Gijbels and Wang (1993) and the well-known Cramér–Wold device. We want to derive the asymptotic distribution of the process $\boldsymbol{V_n}(t) = ( V_{n_1}(t), \ldots , V_{n_k}(t))^\top$, for $t \in [a_F,b]$. According to the Cramér–Wold device, the convergence of the *k*-dimensional process is equivalent to the convergence of

$$\begin{aligned} A_n(t) = \sum _{j=1}^k c_j \sqrt{n_j} \left( F_{jn_j}(t) - F_n(t) \right) , \end{aligned}$$

for any $$c_1, \ldots , c_k \in \mathbb {R}$$.

Simple algebraic manipulations yield

$$\begin{aligned} A_n(t) = \sum _{j=1}^k \left( c_j - p_j \sum _{l=1}^k c_l \sqrt{\frac{n_l}{n_j} }\right) \sqrt{n_j} \left( F_{jn_j}(t) - F(t) \right) = \sum _{j=1}^k \tilde{c}_j A_{n_j}(t), \end{aligned}$$

where $\tilde{c}_j = c_j - p_j \sum _{l=1}^k c_l \sqrt{\frac{n_l}{n_j} }$ and $A_{n_j}(t) = \sqrt{n_j} \left( F_{jn_j}(t) - F(t) \right)$. That is, we have a sum of *k* independent empirical processes, whose convergence has already been studied in Gijbels and Wang (1993). It is then immediate that $A_n(t)$ is convergent in the Skorohod space $\mathbb {D}[a_F,b]$. As proved in Gijbels and Wang (1993), the limit process of $A_{n_j}(t)$, say $A_j(t)$, is a centred Gaussian process in $\mathbb {D}[a_F,b]$, whose covariance structure is given by

$$\begin{aligned} \Gamma _j(t,s) = S_j(t)S_j(s) \int _{a}^{t \wedge s} \frac{ dH^1_j(z) }{C_j(z)^2}, \end{aligned}$$

for all $t,s \in [a_F,b]$, and where $S_j(t) = 1 - F_j(t)$. Then, the process $A_n(t)$ converges and, by means of the Cramér–Wold device, so it does the *k*-dimensional stochastic process $\boldsymbol{V_n}(t) = \left( V_{n_1}(t), \ldots , V_{n_k}(t) \right) ^\top$. The limit of the sequence is a *k*-dimensional centred Gaussian process, say $\boldsymbol{V}(t)= \left( V_1(t), \ldots , V_k(t) \right) ^\top$. The zero-mean property of $\boldsymbol{V(t)}$ is immediate from the definition of $V_{n_j}(t)$. Indeed, note that, under $H_0$,

14 $$\begin{aligned} V_{n_j}(t) = \sqrt{n_j} \left( F_{jn_j}(t) - F(t) - \sum _{j=1}^k p_l \left( F_{n_l}(t) - F(t) \right) \right) , \end{aligned}$$

for all $j \in \{1, \ldots , k\}$ and all $t \in [a_F,b]$. Corollary 1 in Gijbels and Wang (1993) yields that $\mathbb {E}\left[ V_{n_j}(t)\right] =0$.

Rearranging the expression in (14) yields

$$\begin{aligned} V_{n_j}(t) = (1-p_j) \sqrt{n_j} \left( F_{jn_j}(t) - F(t) \right) - \sum _{j=1}^k p_l \sqrt{ \frac{n_j}{n_l} } \sqrt{n_l} \left( F_{n_l}(t) - F(t) \right) . \end{aligned}$$

Let us compute the covariance between $V_{n_j}(t)$ and $V_{n_l}(s)$, for $j,l \in \{1, \ldots , k\}$ and $t,s\in [a_F,b]$. Let us first suppose that $j = l$. In this case, by using the covariance expression in Corollary 1 of Gijbels and Wang (1993),

$$\begin{aligned} Cov( V_{n_j}(t), V_{n_j}(s)) & = (1-p_j)^2 \Gamma _j(t,s) + \sum _{m \not = j} p_m^2 \frac{n_j}{n_m} \Gamma _m(t,s) \\ & \quad = (1-2 p_j) \Gamma _j(t,s) + \sum _{m=1}^k p_m^2 \frac{n_j}{n_m} \Gamma _m(t,s). \end{aligned}$$

Applying that $n_j/n \longrightarrow \pi _j \in (0,1)$, as $n_1, \ldots , n_k \longrightarrow \infty$, for every $j \in \{1, \ldots , k\}$, we obtain

15 $$\begin{aligned} Cov( V_{n_j}(t), V_{n_j}(s)) \longrightarrow (1-2 p_j) \Gamma _j(t,s) + \sum _{m=1}^k p_m^2 \frac{\pi _j}{\pi _m} \Gamma _m(t,s) \end{aligned}$$

as $n_1, \ldots , n_k \longrightarrow \infty$. Let us now address the case $j \not = l$. We write

$$\begin{aligned} Cov( V_{n_j}(t), V_{n_l}(s))= & -(1-p_j)p_j \sqrt{\frac{n_l}{n_j}} \Gamma _j(t,s) -(1-p_l)p_l \sqrt{\frac{n_j}{n_l}} \Gamma _l(t,s) \\ & \quad + \sum _{m \not = j,l} p_m^2 \sqrt{ \frac{n_j n_l}{n_m^2} } \Gamma _m(t,s) \\= & - p_j \sqrt{\frac{n_l}{n_j}} \Gamma _j(t,s) - p_l \sqrt{\frac{n_j}{n_l}} \Gamma _l(t,s) + \sum _{m=1}^k p_m^2 \frac{n_j n_l}{n_m^2} \Gamma _m(t,s). \end{aligned}$$

Using again that $n_j/n \longrightarrow \pi _j \in (0,1)$, as $n_1, \ldots , n_k \longrightarrow \infty$, for every $j \in \{1, \ldots , k\}$, we get

16 $$\begin{aligned} Cov( V_{n_j}(t), V_{n_l}(s)) \longrightarrow \sum _{m=1}^k p_m^2 \frac{\pi _j \pi _l}{\pi _m^2} \Gamma _m(t,s) - p_j \sqrt{\frac{\pi _l}{\pi _j}} \Gamma _j(t,s) - p_l \sqrt{\frac{\pi _j}{\pi _l}} \Gamma _l(t,s), \end{aligned}$$

as $n_1, \ldots , n_k \longrightarrow \infty$. The proof is then concluded. $\square$

### Proof of Corollary2

Let us derive the asymptotic distribution of $\int V_{n_j}(z)^2 dF_n(z)$. On the one hand, note that

$$\begin{aligned} F_n(t) - F(t) = \sum _{j=1}^k p_j ( F_{n_j}(t) - F(t) ). \end{aligned}$$

So, following Corollary 1 in Gijbels and Wang (1993), we get that

17 $$\begin{aligned} F_n(t) - F(t) = o(1) \; \text{ a.s. } \end{aligned}$$

uniformly in $t \in [a_F,b]$. Analogously, by Lemma 1.9.3 and Theorem 1.10.3 in van der Vaart and Wellner (1996), it holds that

18 $$\begin{aligned} \sup _{a_F \le t \le b} \mid V_{n_j}(t) - V_j(t) \mid = o(1) \; \text{ in } \text{ probability}. \end{aligned}$$

On the other hand, let us consider the following decomposition

$$\begin{aligned} \int V_{n_j}(z)^2 dF_n(z) & = \int \left( V_{n_j}(z)^2 - V_j(z)^2 \right) dF_{n}(z) \\ & \quad + \int V_j(z)^2 d \left( F_n(z) - F(z) \right) + \int V_j(z)^2 dF(z). \end{aligned}$$

First, it holds that

19 $$\begin{aligned} \left| \int \left( V_{n_j}(z)^2 - V_j(z)^2 \right) dF_{n}(z) \right| \le \sup _{a_F \le t \le b} \mid V_{n_j}(t) - V_j(t) \mid \sup _{a_F \le t \le b} \mid V_{n_j}(t) + V_j(t) \mid \int dF_{n}(z). \end{aligned}$$

The convergence of $V_{n_j}(t)$ and $V_j(t)$ being a Gaussian process yield that

$$\begin{aligned} \sup _{a_F \le t \le b} \mid V_{n_j}(t) + V_j(t) \mid = O(1) \; \text{ in } \text{ probability}. \end{aligned}$$

This fact, combined with (18) and (19), yields

$$\begin{aligned} \int \left( V_{n_j}(z)^2 - V_j(z)^2 \right) dF_{n}(z) = o(1) \; \text{ in } \text{ probability. } \end{aligned}$$

Moreover, note that $V_j(t)$ is bounded and continuous a.s. Then, by (17) and Theorem 5.2 in Billingsley (1968), we get that

$$\begin{aligned} \int V_j(z)^2 d \left( F_{n_j}(z) - F(z) \right) = o(1) \; \text{ in } \text{ probability. } \end{aligned}$$

An immediate application of Slutsky’s theorem yields

$$\begin{aligned} \int V_{n_j}(z)^2 dF_n(z) \longrightarrow \int V_j(z)^2 dF(z), \end{aligned}$$

in distribution as $n_1, \ldots , n_k \longrightarrow \infty$. $\square$

### Proof of Theorem 2

The proof relies on Corollary 1 in Gijbels and Wang (1993), which states, among other results, that $\sqrt{n_j} \left( F_{jn_j}(t) - F_j(t) \right)$ is bounded a.s., for $j \in \{1, \ldots , k\}$. First, for $j \in \{1, \ldots , k\}$, note that

20 $$\begin{aligned} \nonumber \sqrt{n_j}\left( F_{jn_j}(t) - F_n(t) \right)= & (1-p_j)\sqrt{n_j}\left( F_{jn_j}(t) - F_j(t)\right) - \sum _{l \not = j} p_l \sqrt{ \frac{n_j}{n_l} } \sqrt{n_l}\left( F_{n_l}(t) - F_l(t)\right) \\ & + \sqrt{n_j} \left( F_j(t) - \sum _{l=1}^k p_l F_l(t) \right) . \end{aligned}$$

By Corollary 1 in Gijbels and Wang (1993), $\sqrt{n_l}\left( F_{n_l}(t) - F_l(t)\right) = O(1)$ in probability. Moreover, by hypothesis, for $j,l \in \{1, \dots , k\}$, $n_j/n_l \longrightarrow \pi _j/\pi _l \in (0,\infty )$. As a consequence, the first two terms in the decomposition (20) are bounded in probability. Furthermore, $F_j(t) - \sum _{l=1}^k p_l F_l(t) \not = 0$, since under $H_1$ there exists $m \in \{1, \ldots , k\}$, with $m \not = j$, such that $F_j \not = F_m$ and $p_l >0$, for all $l \in \{1, \ldots , k\}$. We may then conclude that, as $n_1, \ldots , n_k \rightarrow \infty$,

$$\begin{aligned} \sup _{ t \in [\tilde{a}_F,b]} \bigg \vert \sqrt{n_j}\left( F_{jn_j}(t) - F_n(t) \right) \bigg \vert \longrightarrow \infty , \end{aligned}$$

in probability, for all $j \in \{1, \ldots , k\}$.

On the one hand, regarding $D_{KS}$, we may conclude that

$$\begin{aligned} D_{KS}= & \sup _{ t \in [\tilde{a}_F,b]} \sum _{j=1}^k \left( \sqrt{n_j}\left( F_{jn_j}(t) - F_n(t) \right) \right) ^2 \ge \sup _{ t \in [\tilde{a}_F,b]} \left( \sqrt{n_1}\left( F_{1n_1}(t) - F_n(t) \right) \right) ^2 \\= & \left( \sup _{ t \in [\tilde{a}_F,b]} \bigg \vert \sqrt{n_1} \left( F_{1n_1}(t) - F_n(t) \right) \bigg \vert \right) ^2 \longrightarrow \infty , \end{aligned}$$

in probability as $n_1, \ldots , n_k \rightarrow \infty$.

On the other hand, following (20), each integral in $D_{CvM}$ can be written as

$$\begin{aligned} n_j \int _{\tilde{a}_F}^{b} \left( F_{jn_j}(t) - F_n(t) \right) ^2 dF_n(t)= & I_{1j} + I_{2j} + I_{3j}, \end{aligned}$$

where

$$\begin{aligned} I_{1j} = \int _{\tilde{a}_F}^{b} \left( (1-p_j)\sqrt{n_j}\left( F_{jn_j}(t) - F_j(t)\right) - \sum _{l \not = j} p_l \sqrt{ \frac{n_j}{n_l} } \sqrt{n_l}\left( F_{n_l}(t) - F_l(t)\right) \right) ^2 dF_n(t), \end{aligned}$$

$$\begin{aligned} I_{2j} & = 2 \sqrt{n_j} \int _{\tilde{a}_F}^{b} \left( (1-p_j)\sqrt{n_j}\left( F_{jn_j}(t) - F_j(t)\right) - \sum _{l \not = j} p_l \sqrt{ \frac{n_j}{n_l} } \sqrt{n_l}\left( F_{n_l}(t) - F_l(t)\right) \right) \\ & \quad \left( F_j(t) - \sum _{l=1}^k p_l F_l(t) \right) dF_n(t) \end{aligned}$$

and

$$\begin{aligned} I_{3j} = n_j \int _{\tilde{a}_F}^{b} \left( F_j(t) - \sum _{l=1}^k p_l F_l(t) \right) ^2 dF_n(t). \end{aligned}$$

Following Corollary 1 in Gijbels and Wang (1993) and the fact that $n_j/n_l \longrightarrow \pi _j/\pi _l$ for all $j,l \in \{1, \ldots , k\}$, $I_{1j}$ is bounded in probability. By that same result, the integral in $I_{2j}$ is also bounded in probability, thus $I_{2j}$ tends to infinity, in absolute value, at rate $\sqrt{n_j}$. Finally, under $H_1$, it is clear that $I_{3j}$ tends to positive infinity at rate $n_j$. Since $I_{3j}$ is the dominant term, it holds that $D_{CvM} \longrightarrow \infty$, in probability as $n_1, \ldots , n_k \rightarrow \infty$. $\square$

### Proof of Theorem 3

The proof of this result follows the lines of the proof of Theorem 1. There are, however, some remarks that need to be noted for the proof to be correct. Let us consider $V_{n_j}^{b}(t) = \sqrt{n_j} \left( F_{jn_j}^{b}(t) - F_n^{b}(t) \right)$ and construct the *k*-dimensional process $\boldsymbol{V_n^{b}}(t) = \left( V_{n_1}^{b}(t), \ldots , V_{n_k}^{b}(t) \right) ^\top$. To see that the previous *k*-dimensional process converges, by means of the Cramér–Wold device, it is enough to show that

$$\begin{aligned} A_{n}^{b}(t) = \sum _{j=1}^k c_j V_{n_j}^{b}(t) \end{aligned}$$

converges, for every $c_1, \ldots , c_k \in \mathbb {R}$. Note that

$$\begin{aligned} \sqrt{n_j}\left( F_{jn_j}^{b}(t) - F_n^{b}(t) \right) & = (1-p_j)\sqrt{n_j}\left( F_{jn_j}^{b}(t) - F_n(t) \right) \\ & \quad - \sum _{l\not =j} p_l \sqrt{\frac{n_j}{n_l}} \sqrt{n_l}\left( F_{n_l}^{b}(t) - F_n(t) \right) . \end{aligned}$$

Analogously to the proof of Theorem 1, $A_n^{b}(t)$ can be written as

$$\begin{aligned} A_n^{b}(t) = \sum _{j=1}^k \left( c_j - p_j \sum _{l=1}^k c_l \sqrt{\frac{n_l}{n_j} }\right) \sqrt{n_j} \left( F_{jn_j}^{b}(t) - F_n(t) \right) = \sum _{j=1}^k \tilde{c}_j A_{n_j}^{b}(t), \end{aligned}$$

where $\tilde{c}_j = c_j - p_j \sum _{l=1}^k c_l \sqrt{\frac{n_l}{n_j}}$ and $A_{n_j}^{b}(t) = \sqrt{n_j} \left( F_{jn_j}^{*b}(t) - F_n(t) \right)$. That is, $A_n^{b}(t)$ can be written as the sum of independent convergent processes, which ensures the convergence of $A_n^{b}$ and thus the convergence of $\boldsymbol{V_n^{b}}(t)$. The independence can be guaranteed by noting that, conditionally to the original *k* samples, $F_n$ is not random. The proof concludes by following the lines of the proof of Theorem 1 and by means of Theorem 1 in Bilker and Wang (1997). $\square$

## References

[CR1] Asgharian M, M’Lan CE, Wolfson DB (2002) Length-biased sampling with right censoring: an unconditional approach. J Am Stat Assoc 97:201–209

[CR2] Austin M, Betensky R (2014) Eliminating bias due to censoring in Kendall’s tau estimators for quasi-independence of truncation and failure. Comput Stat Data Anal 73:16–26 24505164 10.1016/j.csda.2013.11.018 PMC3912250

[CR3] Bilker WB, Wang MC (1997) Bootstrapping left-truncated and right-censored data. Commun Stat Simul Comput 26:141–171

[CR4] Billingsley P (1968) Convergence of Probability Measures. John Wiley & Sons

[CR5] Birnbaum ZW, Hall RA (1960) Small sample distributions for multi-sample statistics of the Smirnov type. Ann Math Stat 31:710–720

[CR6] Chao MT, Lo SH (1988) Some representations of the nonparametric maximum likelihood estimators with truncated data. Ann Stat 16:661–668

[CR7] Chiou S, Qian J (2025) tranSurv: Transformation-Based Regression under Dependent Truncation. https://doi.org/10.32614/CRAN.package.tranSurv, https://CRAN.R-project.org/package=tranSurv, R package version 1.2.4

[CR8] Conover WJ (1965) Several k-sample Kolmogorov-Smirnov tests. Ann Math Stat 36:1019–1026

[CR9] Efron B (1981) Censored data and the bootstrap. J Am Stat Assoc 76:312–319

[CR10] Giacomini R, Politis DN, White H (2013) A warp-speed method for conducting Monte Carlo experiments involving bootstrap estimators. Economet Theor 29:567–589

[CR11] Gijbels I, Wang JL (1993) Strong representations of the survival function estimator for truncated and censored data with applications. J Multivar Anal 47:210–229

[CR12] Gross ST, Lai TL (1996a) Bootstrap methods for truncated and censored data. Statistica Sinica 6: 509–530

[CR13] Gross ST, Lai TL (1996b) Nonparametric estimation and regression analysis with left-truncated and right-censored data. Journal of the American Statistical Association 91:1166–1180

[CR14] Hyde J (1977) Testing survival under right censoring and left truncation. Biometrika 64:225–230

[CR15] Kaplan EL, Meier P (1958) Nonparametric estimation from incomplete observations. J Am Stat Assoc 53:457–481

[CR16] Kiefer J (1959) K-sample analogues of the Kolmogorov-Smirnov and Cramér-von Mises tests. Ann Math Stat 30:420–447

[CR17] Klein JP, Moeschberger ML (2003) Survival Analysis: Techniques for Censored and Truncated Data. Springer, New York

[CR18] Koziol JA, Green SB (1976) A Cramér-von Mises statistic for randomly censored data. Biometrika 63:465–474

[CR19] Lagakos SW, Barraj LM, de Gruttola V (1988) Nonparametric analysis of truncated survival data, with application to AIDS. Biometrika 75:515–523

[CR20] Lago A, Pardo-Fernández JC, de Uña-Álvarez J, Van Keilegom I (2025a) Density-based tests for the k–sample problem with left-truncated data. Manuscript under review 10.1007/s10985-026-09713-1 PMC13179922 42142198

[CR21] Lago A, de Uña-Álvarez J, Pardo-Fernández JC (2025b) A Kolmogorov-Smirnov-type test for the two-sample problem with left-truncated data. TEST 34:69–90 10.1007/s10985-026-09713-1 PMC13179922 42142198

[CR22] Lai TL, Ying Z (1991) Estimating a distribution function with truncated and censored data. Ann Stat 19:417–442

[CR23] Lynden-Bell D (1971) A method of allowing for known observational selection in small samples applied to 3cr quasars. Mon Not R Astron Soc 155:95–118

[CR24] Mantel N (1966) Evaluation of survival data and two new rank order statistics arising in its consideration. Cancer Chemother Rep 50:163–170 5910392

[CR25] Martin EC, Betensky RA (2005) Testing quasi-independence of failure and truncation times via conditional Kendall’s tau. J Am Stat Assoc 100:484–492

[CR26] Martínez-Camblor P, de Uña-Álvarez J (2009) Non-parametric k-sample tests: Density functions vs distribution functions. Comput Stat Data Anal 53:3344–3357

[CR27] Neuhaus G (1993) Conditional rank tests for the two-sample problem under random censorship. Ann Stat 21:1760–1779

[CR28] Ning J, Qin J, Shen Y (2010) Non-parametric tests for right-censored data with biased sampling. J R Stat Soc Ser B Stat Methodol 72:609–630 10.1111/j.1467-9868.2010.00742.x PMC2963462 21031144

[CR29] Peto R, Peto J (1972) Asymptotically efficient rank invariant test procedures. J Roy Stat Soc Series A General 135:185–198

[CR30] Qian J, Betensky RA (2014) Assumptions regarding right censoring in the presence of left truncation. Stat Probab Lett 87:12–17 24683283 10.1016/j.spl.2013.12.016 PMC3964676

[CR31] R Core Team (2024) R: A Language and Environment for Statistical Computing. R Foundation for Statistical Computing, Vienna, Austria, https://www.R-project.org/

[CR32] Schumacher M (1984) Two-sample tests of Cramér-von Mises and Kolmogorov-Smirnov-type for randomly censored data. Int Stat Rev 52:263–281

[CR33] Shen PS (2003) The product-limit estimate as an inverse-probability-weighted average. Commun Stat Theory Method 32:1119–1133

[CR34] Shen PS (2005) Estimation of the truncation probability with left-truncated and right-censored data. Nonparametric Stat 17:957–969

[CR35] Smirnov NV (1939) On the estimation of the discrepancy between empirical curves of distribution for two independent samples. Mosc Univ Math Bull 2:3–14

[CR36] Stephens M (1974) EDF statistics for goodness of fit and some comparisons. J Am Stat Assoc 69:730–737

[CR37] Strzałkowska-Kominiak E, Stute W (2010) On the probability of holes in truncated samples. J Stat Plann Inf 140:1519–1528

[CR38] Stute W, Wang JL (2008) The central limit theorem under random truncation. Bernoulli 14:604–622 22844204 10.3150/07-BEJ116 PMC3404856

[CR39] Tassie JM, Grabar S, Lancar R, Deloumeaux J, Bentata M, Costagliola D. from the French Hospital Database on HIV CEG (2002) Time to AIDS from 1992 to 1999 in HIV-1-infected subjects with known date of infection. JAIDS Journal of Acquired Immune Deficiency Syndromes 30:81–87 10.1097/00042560-200205010-00011 12048367

[CR40] Therneau T (2026) A Package for Survival Analysis in R. R package version 3.8-6

[CR41] Therneau T, Grambsch P (2000) Modeling Survival Data: Extending the Cox Model. Springer

[CR42] Tsai WY (1990) Testing the assumption of independence of truncation time and failure time. Biometrika 77:169–177

[CR43] Tsai WY, Jewell NP, Wang MC (1987) A note on the product-limit estimator under right censoring and left truncation. Biometrika 74:883–886

[CR44] de Uña-Álvarez J, Iglesias-Pérez MC (2010) Nonparametric estimation of a conditional distribution from length-biased data. Ann Inst Stat Math 62:323–341

[CR45] van der Vaart A, Wellner JA (1996) Weak Convergence and Empirical Processes. Springer, New York

[CR46] Wang MC (1991) Nonparametric estimation from cross-sectional survival data. J Am Stat Assoc 86:130–143

[CR47] Woodroofe M (1985) Estimating a distribution function with truncated data. Ann Stat 13:163–177

[CR48] Zhang J, Wu Y (2007) k-Sample tests based on the likelihood ratio. Comput Stat Data Anal 51:4682–4691

[CR49] Zhou Y, Yip PSF (1999) A strong representation of the product-limit estimator for left truncated and right censored data. J Multivar Anal 69:261–280

